# Amide Hydrogen–Deuterium Exchange in Isotopically Mixed Water

**DOI:** 10.1021/acsphyschemau.5c00127

**Published:** 2026-03-17

**Authors:** Antonio Grimaldi, Billy Hobbs, Michele Stofella, Theodoros K. Karamanos, Emanuele Paci

**Affiliations:** † Department of Physics and Astronomy, 9296 University of Bologna, Bologna 40127, Italy; ‡ Department of Life Sciences, 4615 Imperial College London, London SW7 2AZ, U.K.

**Keywords:** proteins, hydrogen−deuterium exchange, water mixtures, isotope effects, fractionation, chemical kinetics

## Abstract

Hydrogen–deuterium exchange (HDX) of protein backbone amides provides a powerful probe of conformational dynamics. However, when experiments are performed in H_2_O/D_2_O mixtures, quantitative interpretation is hindered by back exchange and isotope effects not captured by the classical Linderstro̷m–Lang (LL) model. We introduce a generalized Linderstro̷m–Lang (GLL) framework that explicitly accounts for forward and reverse exchange and for changes in protection upon isotopic substitution. Analytical solutions describe equilibrium enrichment (fractionation) and protection factors in mixtures, reducing to the LL model in pure D_2_O. Application to HDX/NMR of the molecular chaperone DNAJB1 in 50% D_2_O demonstrates that the GLL model recovers protection factors at 100% D_2_O. Ignoring back exchange (i.e., using the LL model), protection factors are systematically underestimated. A particularly powerful feature of our approach is that a single HDX experiment in a mixture (e.g., 50% D_2_O) simultaneously provides protection factors that report on conformational dynamics and local stability and fractionation factors that are sensitive to the local hydrogen-bonding environment.

## Graphical abstract

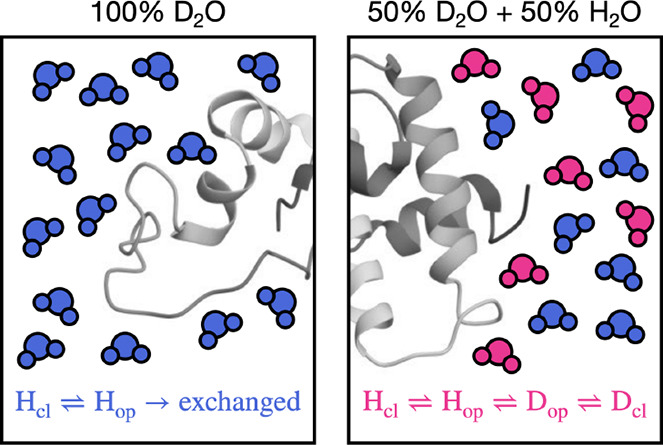

## Introduction

Hydrogen–deuterium exchange (HDX) is a spontaneous process in which the hydrogen atoms of a solute molecule are replaced with deuterium from solvent. HDX in proteins was pioneered by Linderstro̷m-Lang and co-workers^1–3^ with the goal of measuring the exchange rates of backbone amides, which depend on protein primary and higher-order structure, conformational dynamics as well as physicochemical properties of the solvent.^4–7^ Intrinsic HDX rates, that pertain to maximally solvated amides, have been measured in pure H_2_O and D_2_O.^8–10^ Observed HDX rates can be orders of magnitude lower than intrinsic ones, e.g. for amides engaged in stabilizing intramolecular hydrogen bonds or buried in a hydrophobic core. Effects of this kind, collectively termed protection,^4,5^ make HDX-based techniques suitable for fingerprinting protein structure and dynamics, with applications spanning the study of folding^11^ and allostery^12^ to protein–ligand interactions, and the development of novel therapeutics.^13,14^ HDX data can aid prediction of protein conformational ensembles^15^ and understanding of intrinsically disordered proteins.^16^

The Linderstro̷m-Lang (LL) model^4^ describes HDX of proteins in pure D_2_O. It assumes that each backbone amide hydrogen adopts either closed (H_cl_) or open (H_op_) conformations, only the latter being competent to exchange according to the reaction

$$H_{\mathrm{cl}}\overset{k_{\mathrm{op}}}{\underset{k_{\mathrm{cl}}}{⇌}}H_{\mathrm{op}}\overset{k_{\mathrm{int}}}{\to }\mathrm{exchanged}$$ 1

The rate constants of opening and closing transitions, *k*_op_ and *k*_cl_, encode protein conformational dynamics. Their ratio is the protection factor *P* = *k*_cl_/*k*_op_, which is the reciprocal of the opening equilibrium constant, related to the opening free energy Δ*G*_op_ = *G*_op_ – *G*_cl_ by^6^

$$\Delta G_{\mathrm{op}}=RT\;\ln\;P$$ 2

The intrinsic exchange rate *k*_int_ of an amide depends on neighboring residues, temperature and pH.^8,10^ At near-neutral pH, the conditions *k*_cl_ ≫ *k*_op_ (*P* ≫ 1) and *k*_cl_ ≫ *k*_int_ are satisfied by most amides of native proteins, and exchange occurs in the so-called EX2 limit.^4^ In this case, the exchanged fraction of initially undeuterated amides is given by a single exponential

$$D\left(t\right)=1-e^{-k_{\mathrm{obs}}t}$$ 3

with observed rate constant

$$k_{\mathrm{obs}}=\frac{k_{\mathrm{int}}}{P}$$ 4

A number of analytical methods sensitive to the properties of hydrogen isotopes can detect exchange.^17^ The experiment (or steps thereof) is often performed in H_2_O/D_2_O mixtures. The LL model ([Disp-formula eq1]) does not account for the back exchange and isotope effects observed in mixtures. A generalized theoretical framework is developed here that incorporates these effects as an extension to the LL model, also opening the door to understanding (and, crucially, correcting for) back-exchange in HDX/MS workflows.^18,19^ The model is applied to HDX/NMR measurements performed in 50% and pure D_2_O. The construct used in this study was the 112-residue (12.4 kDa) JD-GF-α5 F94L variant of DNAJB1, a class B J-domain protein (Hsp40) comprising a well-folded N-terminal J-domain (residues 1–69), followed by a largely disordered glycine/phenylalanine-rich linker (residues 70–96) and an autoinhibitory helix, α5 (residues 96–106).^20^ The F94L mutation in the linker region has recently been shown to weaken this autoinhibitory interaction.^21^ The mixed folded nature of this construct gives rise to a large dynamic range of solvent exchange rates with residues in the linker, α5 and those in the loops connecting helices 2 and 3 of the J-domain showing exchange rates in the ms time scale. Therefore, only residues in the well-folded J-domain were able to be probed by the HDX experiment. Protection factors extracted from the experiment in the mixture using the generalized model are consistent with the results for pure D_2_O analyzed using the LL model. An extra piece of information, solely available from measurements in mixtures, is the fractionation that reports on the local hydrogen bonding network^22–27^ and may complement the protection factors in modeling structural ensembles.

## Theoretical Framework

Amide HDX in a H_2_O/D_2_O mixture can be described by the generalized Linderstro̷m-Lang (GLL) model

$$H_{\mathrm{cl}}\overset{k_{\mathrm{op}}}{\underset{k_{\mathrm{cl}}}{⇌}}H_{\mathrm{op}}\overset{k_{\mathrm{forw}}}{\underset{k_{\mathrm{back}}}{⇌}}D_{\mathrm{op}}\overset{k_{\mathrm{cl}}^{\prime }}{\underset{k_{\mathrm{op}}^{\prime }}{⇌}}D_{\mathrm{cl}}$$ 5

Open and closed states are defined as for the LL model (1), and their interconversion rates differ upon isotopic substitution, i.e. *k*_cl_ *′* ≠ *k*_cl_ and *k*_op_ *′* ≠ *k*_op_. This implies a different protection factor for the deuterated amide *P′* = *k*_cl_ *′*/*k*_op_ *′*. *P′* can be written as *P′* = *P*(1 + δ), where δ can be positive or negative and determines the difference in opening free energies

$$\Delta \Delta G_{\mathrm{op}}=\Delta G_{\mathrm{op},D}-\Delta G_{\mathrm{op},H}=RT\;\ln\;(1+\delta )$$ 6

where Δ*G*_op,H_ is defined in eq 2 and Δ*G*_op,*D*_ = *RT* ln *P′*. For unprotected amides, exchange occurs with forward and back exchange rate constants *k*_forw_ and *k*_back_ that depend on sequence, temperature and pH analogously to *k*_int_ in the LL model, as well as D_2_O content. Forward and back exchange rate constants can be estimated as described in ref 28. The approach-to-equilibrium rate of the elementary exchange reaction defines an intrinsic HDX rate in the mixture *k*_int,mix_ = *k*_forw_ + *k*_back_, and the equilibrium constant of the back exchange reaction is *K*_back_ = *k*_back_/*k*_forw_. An energy diagram of the GLL model (5) is sketched in Figure 1.

**Figure 1:**
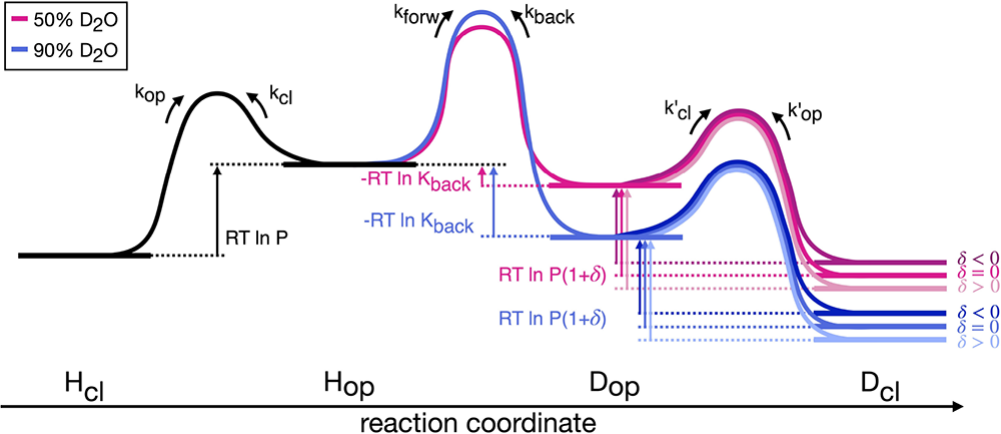
Energy diagram for the GLL model ([Disp-formula eq5]). Exchange in the EX2 limit (high protection and exchange slower than opening/closing dynamics) is considered in two hypothetical mixtures at same pH and temperature and different compositions, 50% (magenta) and 90% (blue) D_2_O. Rate constants *k* ^‡^ are related to the height of the corresponding barriers Δ*G* ^‡^ by the Eyring equation: *k* ^‡^ = (*k*_B_ *T*/*h*)­e^–Δ*G* ^‡^/*RT*^. The solvent composition affects *k*_forw_, *k*_back_ and their ratio *K*_back_, determining the equilibrium between open states. A change in opening free energy upon isotopic substitution that is quantified by δ, *cfr* eq 6, affects equilibrium between deuterated states. The height of the barrier depends on *k*_cl_ *′* and *k*_op_ *′*. In this illustration, it is assumed *k*_cl_ *′* = *k*_cl_ and *k*_op_ *′* varying according to δ.

Rate equations for the GLL model (5) can be exactly solved and converge to a stationary state. Closed-form expressions are obtained in approximations that involve separation of time scales, *cfr* Supporting Information.

The analogous of the EX2 approximation, obtained assuming *P*, *P′* ≫ 1 and *k*_cl_, *k*_cl_ *′* ≫ *k*_int,mix_, gives

$$D\left(t\right)=D_{\mathrm{eq}}+\left(D_{0}-D_{\mathrm{eq}}\right)e^{-k_{\mathrm{obs}}t}$$ 7

where *D*_0_ is the initial condition,

$$D_{\mathrm{eq}}=\frac{1+\delta }{1+\delta +K_{\mathrm{back}}}$$ 8

is the fraction of deuterated amides at equilibrium, and

$$k_{\mathrm{obs}}=\frac{k_{\mathrm{int},\mathrm{mix}}}{P}\left(1-\frac{K_{\mathrm{back}}}{1+K_{\mathrm{back}}}\frac{\delta }{1+\delta }\right)$$ 9

From eqs 8 and 9, it results that back exchange and the difference in protection upon deuteration determine equilibrium and kinetics of HDX reactions. In a mixture with D_2_O mole fraction *x* (H_2_O mole fraction 1 – *x*), in general *D*_eq_ ≠ *x*. The equilibrium ratio

$$\frac{D_{\mathrm{eq}}}{H_{\mathrm{eq}}}=\frac{1+\delta }{K_{\mathrm{back}}}$$ 10

is related to the fractionation factor

$$\varphi =\frac{D_{\mathrm{eq}}}{H_{\mathrm{eq}}}\frac{1-x}{x}$$ 11

that quantifies amide enrichment in deuterium with respect to the solvent.^29^ If δ = 0, the effect on kinetics amounts to replacing *k*_int_ from eq 4 with *k*_int,mix_ that accounts for simultaneous forward and reverse exchange. In the broader case δ ≠ 0, an additional term that depends on both δ and *K*_back_ appears, *cfr* eq 9. In pure D_2_O, *k*_forw_ = *k*_int_ and *k*_back_ = 0, which imply *k*_int,mix_ = *k*_int_ and *K*_back_ = 0, hence *D*_eq_ = 1, *k*_obs_ = *k*_int_/*P*, and the LL model is recovered.

## Results and Discussion

HDX/NMR measurements of initially undeuterated ^15^N-DNAJB1 JD-GF-α5 F94L^21^ were performed at 25 °C in 50% (pH_read_ = 7.40) and 100% (pH_read_ = 7.47) D_2_O, *cfr* Methods. Results are shown for the 18 J-domain amides whose exchange kinetics are well resolved in both conditions. Faster-exchanging sites (including residues in the linker/α5 region and in J-domain loops) undergo substantial exchange during the acquisition dead time and cannot be fit reliably. The 18 residues reported here are therefore the only sites common to both data sets and all fall in the strongly protected regime, with exchange well described by EX2.

Data points, i.e., peak intensities over time were measured in a series of ^1^H–^15^N SOFAST HMQC spectra and fitted to a single exponential *I*(*t*) = *a*e^–*bt*^ + *c*. The fraction of unexchanged amides at time *t* is 1 – *D*(*t*) = *I*(*t*)/*I*(0), where *I*(0) = *a* + *c* is the extrapolated intensity at *t* = 0. The observed exchange rate is *k*_obs_ = *b*. The equilibrium fractions of deuterated and undeuterated sites are $D_{\mathrm{eq}}=\frac{a}{a+c}$ and $H_{\mathrm{eq}}=\frac{c}{a+c}$ , respectively. Data and fit for residue L11 are shown as an example in Figure 2A. Curves for all measured amides are provided, *cfr* Supporting Information. Protection factors were estimated from measurements in 100% D_2_O using the LL model, that is, by eq 4 (Figure 2B). Fractionation factors (Figure 2C) were derived from the parameters of the fit in 50% D_2_O as φ = *D*_eq_/*H*_eq_ = *a*/*c*.

**Figure 2:**
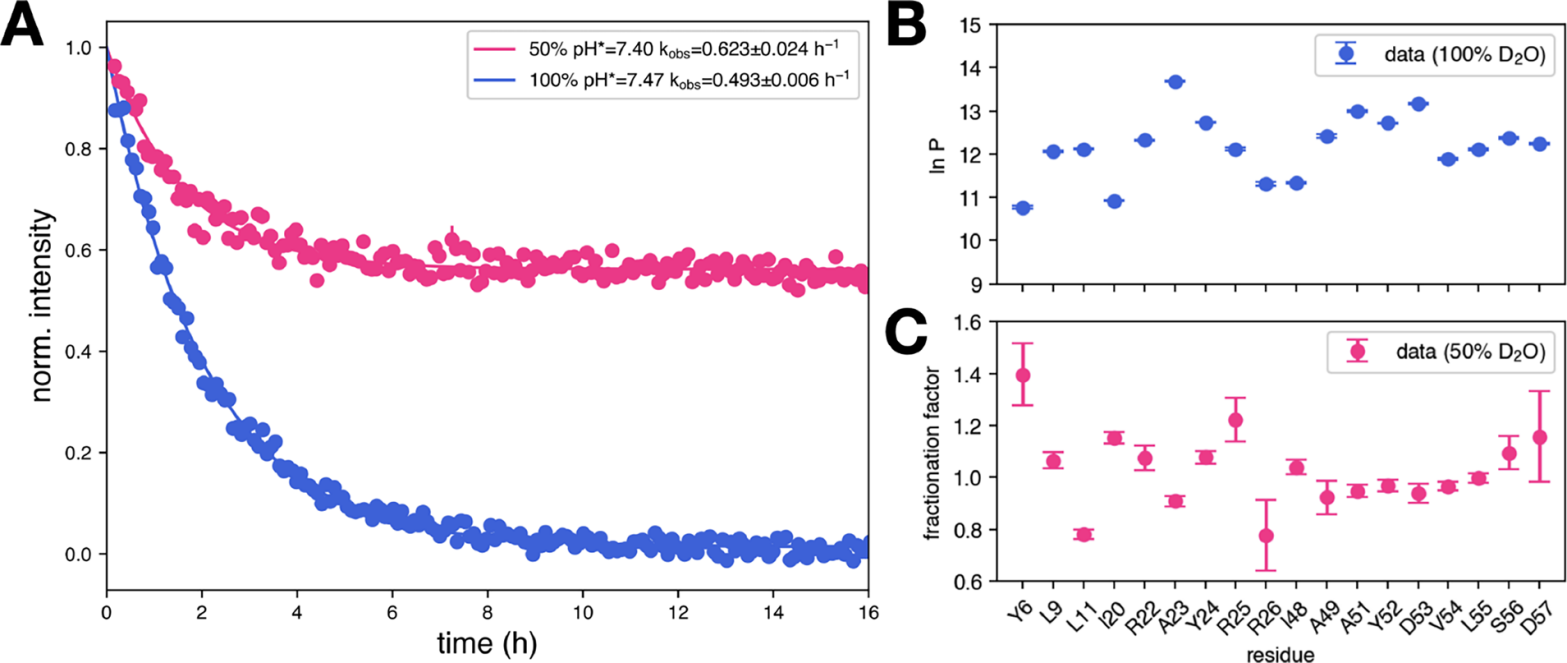
Experimental results for HDX/NMR of ^15^N-DNAJB1 JD-GF-α5 F94L, at 25 °C in 50% D_2_O and pH_read_ = 7.40 and 100% D_2_O and pH_read_ = 7.47. (A) Measured kinetics of residue L11, in 50% (magenta) and 100% (blue) D_2_O. The normalized intensity is the fraction of unexchanged amides, 1 – *D*(*t*). (B) Protection factors (ln*P*) estimated by measurements in 100% D_2_O. (C) Fractionation factors determined from equilibrium values in 50% D_2_O.

For measurements performed in mixtures, *k*_forw_ and *k*_back_ were estimated as a function of temperature, pH and sequence, as described in ref 28. Accordingly, *k*_int,mix_ and *K*_back_ were computed as their sum and their ratio, vide supra. A value of *K*_back_ = 0.83 was consistently found for all residues (this because *K*_back_ refers to the exchange of unprotected amides, for which the model^28^ predicts φ = 1.20, in agreement with reported observations on PDLA^25^). The parameter δ was computed from the measured fractionation φ as δ = *K*_back_φ – 1 (eqs 10 and 11), and directly yields the difference in opening free energy ΔΔ*G*_op_ resulting from isotopic substitution (eq 6), shown in Figure 3A. Finally, protection factors were estimated using the GLL model (5) in EX2 approximation (eq 9), *cfr* Figure 3B.

**Figure 3:**
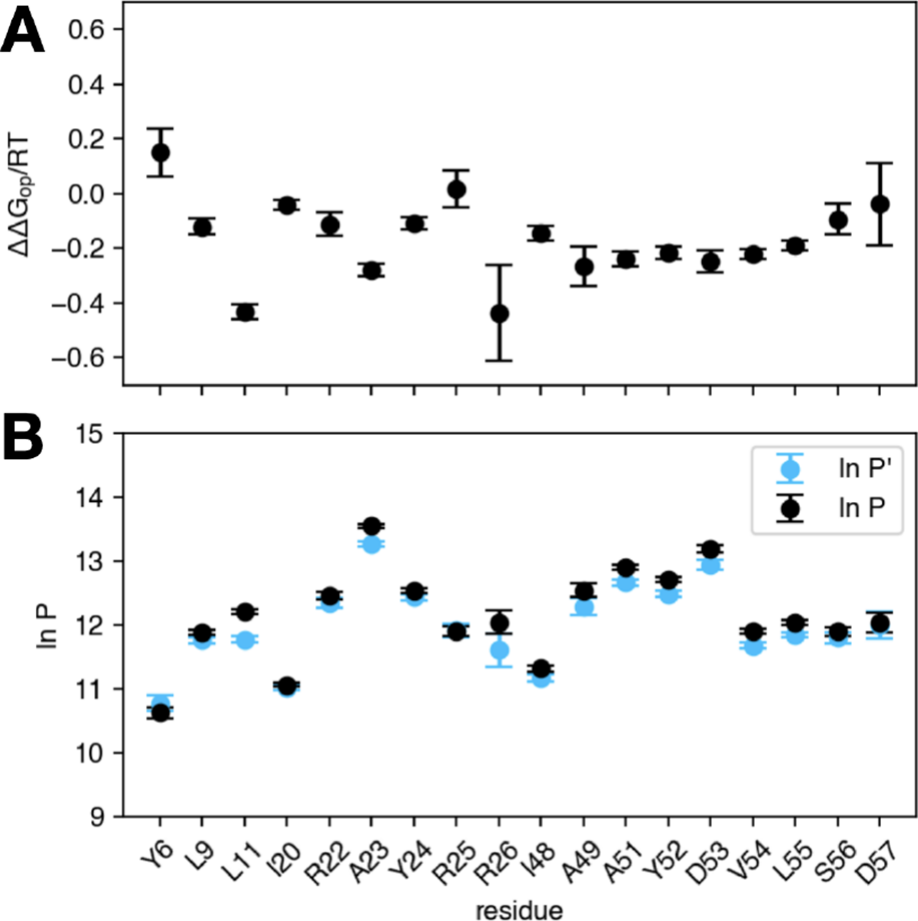
Results obtained by the generalized Linderstro̷m-Lang model for HDX/NMR of ^15^N-DNAJB1 JD-GF-α5 F94L, performed in 50% D_2_O. (A) Difference in local stability (protection factors) upon isotopic substitution quantified by ΔΔ*G*_op_. (B) Inferred protection factors for undeuterated (ln*P*) and deuterated (ln*P′*) amides.

Figure 4A,B display HDX curves for residues L11 and Y6 fitted by the GLL model. In the former, a reduction in local stability upon isotope substitution (δ < 0) causes an upward shift of the plateau, i.e., favors retention of H isotope. In the latter, a shift in the opposite direction is observed (δ > 0).

**Figure 4:**
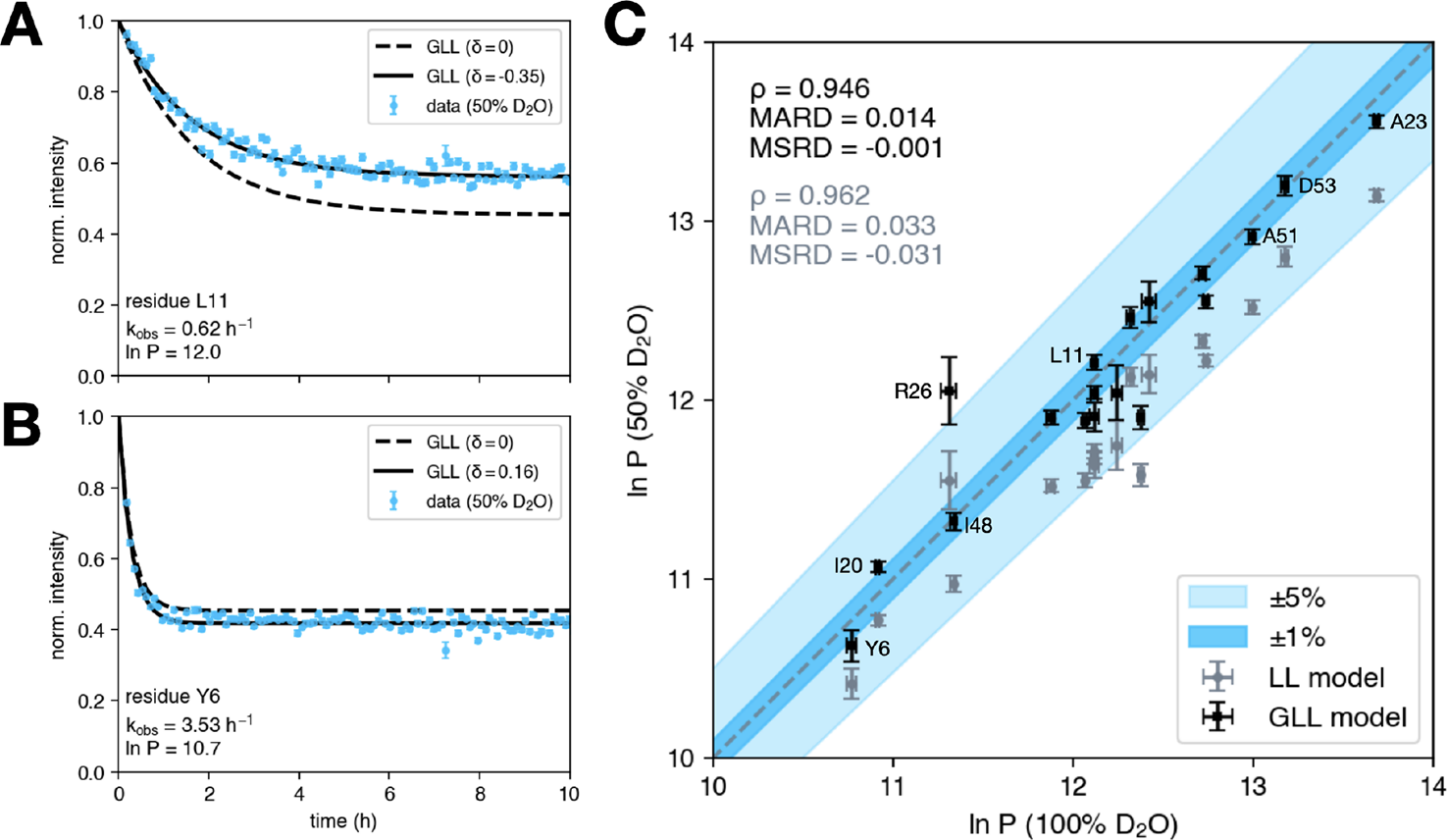
HDX experimental data (dots) obtained from measurements in 50% D_2_O for residues (A) L11 and (B) Y6 and reproduced by the GLL model. Solid lines are generated from the full model, in which δ ≠ 0 indicates variation in local stability upon isotopic substitution and results in fractionation, as well as minor alteration to the kinetics. Dashed lines are obtained considering same *k*_int,mix_ and *P*, and δ = 0. (C) Pair plot of protection factors extracted from data in 100% D_2_O versus protection factors estimated by LL (gray dots) and GLL (black dots) models from data in 50% D_2_O.

The protection factors obtained in 50% D_2_O using the GLL model well reproduce results from pure D_2_O. (Pearson’s ρ = 0.946). To quantify their similarity and bias, one can consider the mean absolute relative deviation (MARD),

$$\mathrm{MARD}=\frac{1}{N}\overset{N}{\underset{i=1}{\sum }}\left|\varepsilon_{i}\right|$$

and the mean signed relative deviation (MSRD),

$$\mathrm{MSRD}=\frac{1}{N}\overset{N}{\underset{i=1}{\sum }}\varepsilon_{i}$$

where

$$\varepsilon_{i}=\frac{\ln\;P_{i,50\%}-\ln\;P_{i,100\%}}{\ln\;P_{i,100\%}}$$

is the relative deviation between protection factors inferred in 50% and 100% D_2_O for the *i*-th measured residue (*i* = 1, 2, ···, *N* = 18). The relative deviation is below 1% for half data points (MARD = 0.014), and presents no bias (MSRD = −0.001). An analogous comparison was made with the protection factors one would recover using the LL formula (eq 4), as a function of the observed exchange rate only, and considering the intrinsic rate in pure D_2_O. In this case, more substantial MARD (= 0.033) and MSRD (= −0.031) suggest that the LL formula systematically underestimates protection factors. Results from both methods are shown in Figure 4C. Residue R26 shows the largest discrepancy, for which a larger ln*P* is predicted in 50% D_2_O. One possible reason is a poor fitting of the rates or the extrapolated intensity, due to considerable exchange occurred before the earliest time point. A second one is a genuine solvent isotope effect on local stability: replacing H_2_O with D_2_O can alter protein thermodynamics in some cases, potentially making *P* weakly dependent on solvent composition.^30,31^ The close agreement for the other residues suggests this effect is small under the different conditions examined here, but R26 serves as a practical reminder that solvent-dependent stability should be considered when relating parameters extracted from a pure solvent and a mixture.

The LL provides straightforwardly (from eq 4) protection factors from exchange measurement in 100% D_2_O (Figure 2B). The GLL model presented here simultaneously provides two parameters: protection factors and fractionation factors from a single HDX experiment in a mixture (e.g., 50% D_2_O). As protection has been related to burial and involvement of amides in hydrogen bonds,^15,32–35^ fractionation has been related to strength of hydrogen bonds in that that low fractionation correlates with and strong hydrogen bonds.^22–27^ A moderate anticorrelation was found between ln *P* from pure D_2_O and the values of ΔΔ*G*_op_ inferred from the experiment in the mixture, *cfr* Figure S2. The values of ΔΔ*G*_op_ vary in fairly broad ranges, and do not appear strongly biased depending on the helix they belong to, or the residue type (with wider range observed for polar amino acids), *cfr* Figure S3. While a rigorous microscopic determinants of protection and fractionation factors is still lacking, it is stressed here that both quantities are rigorously defined and measurable important local parameters that characterize the complex dynamical properties of a protein.

## Methods

Samples of ^15^N-DNAJB1 JD-GF-α5 F94L were expressed and purified as described previously.^21^ In particular, samples were prepared in 20 mM sodium phosphate pH 7.0, 50 mM NaCl and lyophilized. Freeze-dried protein was resuspended in 50 and 100% (v/v) D_2_O, placed into an NMR tube and the loss of intensity of amide protons was monitored using SOFAST ^1^H–^15^N HMQC experiments at 25 °C and 600 MHz. Experiments were recorded with 128 increments in the indirect dimension with 4 scans per increment, 1024 complex points and a D1 of 0.5 s for a total experimental time of about 5 min.

## Supplementary Material



## Notes

Exact solution and approximations for the generalized Linderstro̷m-Lang model, Supporting Figures described in the main text (PDF)

## References

[1] Hvidt A., Linderstro̷m-Lang K. (1954). Exchange of hydrogen atoms in insulin
with deuterium atoms in aqueous solutions. Biochim.
Biophys. Acta.

[2] Linderstro̷m-Lang K. (1955). The pH-dependence
of the deuterium exchange of insulin. Biochim.
Biophys. Acta.

[3] Englander S., Mayne L., Bai Y., Sosnick T. (1997). Hydrogen exchange:
the modern legacy of Linderstro̷m-Lang. Protein science.

[4] Hvidt A., Nielsen S. O. (1966). Hydrogen exchange in proteins. Advances in protein chemistry.

[5] Englander S. W., Kallenbach N. R. (1983). Hydrogen exchange and structural
dynamics of proteins
and nucleic acids. Q. Rev. Biophys..

[6] Hamuro Y. (2021). Tutorial:
chemistry of hydrogen/deuterium exchange mass spectrometry. J. Am. Soc. Mass Spectrom..

[7] James E. I., Murphree T. A., Vorauer C., Engen J. R., Guttman M. (2022). Advances in
hydrogen/deuterium exchange mass spectrometry and the pursuit of challenging
biological systems. Chem. Rev..

[8] Bai Y., Milne J. S., Mayne L., Englander S. W. (1993). Primary
structure effects on peptide group hydrogen exchange. Proteins: Struct., Funct., Bioinf..

[9] Connelly G. P., Bai Y., Jeng M.-F., Englander S. W. (1993). Isotope effects in peptide group
hydrogen exchange. Proteins: Struct., Funct.,
Bioinf..

[10] Nguyen D., Mayne L., Phillips M. C., Walter Englander S. (2018). Reference
parameters for protein hydrogen exchange rates. J. Am. Soc. Mass Spectrom..

[11] Englander S. W., Mayne L., Kan Z.-Y., Hu W. (2016). Protein folding—how
and why: by hydrogen exchange, fragment separation, and mass spectrometry. Annual Review of Biophysics.

[12] Englander S. W. (2023). HX and
Me: Understanding allostery, folding, and protein machines. Annual Review of Biophysics.

[13] Masson G. R., Jenkins M. L., Burke J. E. (2017). An overview
of hydrogen deuterium
exchange mass spectrometry (HDX-MS) in drug discovery. Expert opinion on drug discovery.

[14] Krishnamurthy S., Musgaard M., Tehan B. G., Jazayeri A., Liko I. (2025). The evolving
role of hydrogen/deuterium exchange mass spectrometry in early-stage
drug discovery. Curr. Opin. Struct. Biol..

[15] Devaurs D., Antunes D. A., Borysik A. J. (2022). Computational
modeling of molecular
structures guided by hydrogen-exchange data. J. Am. Soc. Mass Spectrom..

[16] Ghafouri H., Kadeřávek P., Melo A. M., Aspromonte M. C., Bernadó P., Cortes J., Dosztányi Z., Erdos G., Feig M., Janson G. (2025). Towards
a Unified Framework for Determining Conformational Ensembles of Disordered
Proteins. arXiv.

[17] Engen J. R., Wales T. E. (2015). Analytical aspects
of hydrogen exchange mass spectrometry. Annual
Review of Analytical Chemistry.

[18] Stofella M., Grimaldi A., Smit J. H., Claesen J., Paci E., Sobott F. (2024). Computational Tools
for Hydrogen–Deuterium Exchange
Mass Spectrometry Data Analysis. Chem. Rev..

[19] Konermann L., Scrosati P. M. (2024). Hydrogen/Deuterium
Exchange Mass Spectrometry: Fundamentals,
Limitations, and Opportunities. Molecular &
Cellular Proteomics: MCP.

[20] Karamanos T. K., Tugarinov V., Clore G. M. (2019). Unraveling the structure
and dynamics
of the human DNAJB6b chaperone by NMR reveals insights into Hsp40-mediated
proteostasis. Proc. Natl. Acad. Sci. U. S. A..

[21] Hobbs B., Limmer N., Ossa F., Knüpling E., Lenton S., Foderà V., Kalverda A. P., Karamanos T. K. (2025). A low-complexity
linker as a driver of intra-and intermolecular interactions in DNAJB
chaperones. Nat. Commun..

[22] Cleland W., Kreevoy M. M. (1994). Low-barrier hydrogen bonds and enzymic catalysis. Science.

[23] Loh S. N., Markley J. L. (1994). Hydrogen bonding in proteins as studied by amide hydrogen
D/H fractionation factors: application to staphylococcal nuclease. Biochemistry.

[24] Edison A. S., Weinhold F., Markley J. L. (1995). Theoretical studies of protium/deuterium
fractionation factors and cooperative hydrogen bonding in peptides. J. Am. Chem. Soc..

[25] Bowers P. M., Klevit R. E. (1996). Hydrogen bonding and equilibrium isotope enrichment
in histidine-containing proteins. Nat. Struct.
Biol..

[26] LiWang A. C., Bax A. (1996). Equilibrium protium/deuterium fractionation of backbone amides in
U- 13C/15N labeled human ubiquitin by triple resonance NMR. J. Am. Chem. Soc..

[27] Krantz B. A., Moran L. B., Kentsis A., Sosnick T. R. (2000). D/H amide kinetic
isotope effects reveal when hydrogen bonds form during protein folding. Nat. Struct. Biol..

[28] Grimaldi A., Stofella M., Paci E. (2026). Intrinsic Hydrogen Deuterium Exchange
Rates in H2O/D2O Mixtures. J. Phys. Chem. B.

[29] Gold V. (1969). Advances in
Physical Organic Chemistry. Elsevier.

[30] Makhatadze G. I., Clore G. M., Gronenborn A. M. (1995). Solvent
isotope effect and protein
stability. Nat. Struct. Biol..

[31] Giubertoni G., Bonn M., Woutersen S. (2023). D2O as an
imperfect replacement for
H2O: Problem or opportunity for protein research?. J. Phys. Chem. B.

[32] Hilser V. J., Freire E. (1996). Structure-based calculation of the
equilibrium folding
pathway of proteins. Correlation with hydrogen exchange protection
factors. Journal of molecular biology.

[33] Vendruscolo M., Paci E., Dobson C. M., Karplus M. (2003). Rare fluctuations of
native proteins sampled by equilibrium hydrogen exchange. J. Am. Chem. Soc..

[34] Best R. B., Vendruscolo M. (2006). Structural interpretation of hydrogen exchange protection
factors in proteins: characterization of the native state fluctuations
of CI2. Structure.

[35] Skinner J. J., Lim W. K., Bédard S., Black B. E., Englander S. W. (2012). Protein
hydrogen exchange: Testing current models. Protein
Sci..

