# Supplementary material for: Amide Hydrogen–Deuterium Exchange in Isotopically Mixed Water
Source: ACS Phys Chem Au. 2026 Mar 17;6(3):446–50. doi: 10.1021/acsphyschemau.5c00127 (PMC13220189; doi:10.1021/acsphyschemau.5c00127)
Supplement: Supplementary file 1 [file pg5c00127_si_001.pdf]

Supporting Information for
Amide Hydrogen Deuterium Exchange in Isotopically Mixed Water

Antonio Grimaldi,† Billy Hobbs,‡ Michele Stofella,† Theodoros K. Karamanos,‡ and Emanuele Paci*,†

†*Department of Physics and Astronomy, University of Bologna, Bologna, 40127, Italy*

‡*Department of Life Sciences, Imperial College London, London, SW7 2AZ, UK*

E-mail: e.paci@unibo.it

Contents

1 Linderstrøm-Lang model **S2**

1.1 Exact solution S2

1.2 Approximations S3

2 Generalized Linderstrøm-Lang model **S4**

2.1 Exact solution S4

2.2 Approximations S6

2.2.1 Steady state approximation S6

2.2.2 Pre-equilibrium approximation S8

2.2.3 No isotopic substitution effects S9

3 Supporting Figures **S11**

1 Linderstrøm-Lang model

The Linderstrøm-Lang (LL) model[1] describes hydrogen deuterium exchange of proteins in pure D_2O. It assumes that each protein backbone amide hydrogen adopts either closed (H_{cl}) or open (H_{op}) conformations, only the latter being competent to exchange according to the reaction

$$\mathrm{H_{cl}} \underset{k_{\mathrm{cl}}}{\overset{k_{\mathrm{op}}}{\rightleftharpoons}} \mathrm{H_{op}} \xrightarrow{k_{\mathrm{int}}} \text{exchanged.} \tag{S1}$$

The rate constants k_{op} and k_{cl} that are related to opening and closing transitions encode protein dynamics. Their ratio $P = k_{\mathrm{cl}}/k_{\mathrm{op}}$, which is the reciprocal of the opening equilibrium constant, is called the protection factor and is a key quantity to characterize conformational ensembles.[2] The protection factor is related to the opening free energy $\Delta G_{\mathrm{op}} = G_{\mathrm{op}} - G_{\mathrm{cl}}$ by

$$\Delta G_{\mathrm{op}} = RT \ln P. \tag{S2}$$

The intrinsic rate k_{int} is estimated as a function of primary structure, temperature, pH.[3–5]

1.1 Exact solution

The reaction (S1) is associated to a set of coupled linear differential equations:

$$\dot{H}_{\mathrm{cl}}(t) = -k_{\mathrm{op}} H_{\mathrm{cl}}(t) + k_{\mathrm{cl}} H_{\mathrm{op}}(t), \tag{S3a}$$

$$\dot{H}_{\mathrm{op}}(t) = +k_{\mathrm{op}} H_{\mathrm{cl}}(t) - (k_{\mathrm{cl}} + k_{\mathrm{int}}) H_{\mathrm{op}}(t), \tag{S3b}$$

$$\dot{D}(t) = +k_{\mathrm{int}} H_{\mathrm{op}}(t), \tag{S3c}$$

where $H_{\mathrm{cl}}(t)$, $H_{\mathrm{op}}(t)$, $D(t)$ are normalized populations, i.e. their sum is 1. Eqs. S3a, S3b, S3c can be compactly written as $\dot{\mathbf{x}}(t) = \mathbf{K}\mathbf{x}(t)$, where $\mathbf{x}(t) = \begin{bmatrix} H_{\mathrm{cl}}(t) & H_{\mathrm{op}}(t) & D(t) \end{bmatrix}^{\mathrm{T}}$, and

$$\mathbf{K} = \begin{bmatrix} -k_{\mathrm{op}} & +k_{\mathrm{cl}} & 0 \\ +k_{\mathrm{op}} & -k_{\mathrm{cl}} - k_{\mathrm{int}} & 0 \\ 0 & +k_{\mathrm{int}} & 0 \end{bmatrix}. \tag{S4}$$

The solution can be written as

$$\mathbf{x}(t) = \sum_{\lambda} c_{\lambda} \mathrm{e}^{\lambda t} \mathbf{v}_{\lambda},$$

where $\{\lambda\}$ and $\{\mathbf{v}_\lambda\}$ are eigenvalues and eigenvectors of $\mathbf{K}$, while coefficients $\{c_\lambda\}$ depend upon initial conditions. The eigenvectors and eigenvalues are

$$\begin{cases} \lambda_0 = 0 \\ \mathbf{v}_0 = \begin{bmatrix} 0 & 0 & 1 \end{bmatrix}^{\mathrm{T}} \end{cases}, \tag{S5a}$$

$$\begin{cases} \lambda_\pm = -\frac{1}{2}\left((k_{\mathrm{op}} + k_{\mathrm{cl}} + k_{\mathrm{int}}) \pm \sqrt{(k_{\mathrm{op}} + k_{\mathrm{cl}} + k_{\mathrm{int}})^2 - 4k_{\mathrm{op}}k_{\mathrm{int}}} \right) \\ \mathbf{v}_\pm = \begin{bmatrix} \frac{\lambda_\pm}{k_{\mathrm{int}}} - 1 & -\frac{\lambda_\pm}{k_{\mathrm{int}}} & 1 \end{bmatrix}^{\mathrm{T}} \end{cases}. \tag{S5b}$$

1.2 Approximations

The exchanged fraction $D(t)$ is commonly written as a single exponential in the native approximation ($k_{\mathrm{cl}} \gg k_{\mathrm{op}}$):

$$D(t) = 1 - \mathrm{e}^{-k_{\mathrm{obs}}t},$$

where

$$k_{\mathrm{obs}} = \frac{k_{\mathrm{op}}k_{\mathrm{int}}}{k_{\mathrm{cl}} + k_{\mathrm{int}}} \tag{S6}$$

is the observed exchange rate. This expression suggests two limiting cases depending on the relative magnitude of $k_{\mathrm{cl}}, k_{\mathrm{int}}$:

$$\text{EX1} \quad k_{\mathrm{cl}} \ll k_{\mathrm{int}} \qquad k_{\mathrm{obs}} = k_{\mathrm{op}}, \tag{S7a}$$

$$\text{EX2} \quad k_{\mathrm{cl}} \gg k_{\mathrm{int}} \qquad k_{\mathrm{obs}} = \frac{k_{\mathrm{int}}}{P}. \tag{S7b}$$

To avoid the assumption $k_{\mathrm{cl}} \gg k_{\mathrm{op}}$, one possibility is to assume pre-equilibrium between open and closed states whence motility ($k_{\mathrm{op}} + k_{\mathrm{cl}}$) is much faster than exchange (k_{int}). Then,

$$k_{\mathrm{obs}} = \frac{k_{\mathrm{int}}}{1 + P}. \tag{S8}$$

Note that Eq. S8 holds for any P and reduces to the EX2 case (Eq. S7b) if $P \gg 1$, i.e. if the condition $k_{\mathrm{cl}} \gg k_{\mathrm{op}}$ is reintroduced. Conversely, if $P \ll 1$, $k_{\mathrm{obs}} \simeq k_{\mathrm{int}}$.

2 Generalized Linderstrøm-Lang model

The generalized Linderstrøm-Lang (GLL) model

$$\mathrm{H_{cl}} \underset{k_{\mathrm{cl}}}{\overset{k_{\mathrm{op}}}{\rightleftharpoons}} \mathrm{H_{op}} \underset{k_{\mathrm{back}}}{\overset{k_{\mathrm{forw}}}{\rightleftharpoons}} \mathrm{D_{op}} \underset{k'_{\mathrm{op}}}{\overset{k'_{\mathrm{cl}}}{\rightleftharpoons}} \mathrm{D_{cl}}, \tag{S9}$$

is described by a system of ordinary differential equations $\dot{\mathbf{x}}(t) = \mathbf{K}\mathbf{x}(t)$, where $\mathbf{x}(t)$ is the state vector of the system, whose components are the (normalized) populations $H_{\mathrm{cl}}(t)$, $H_{\mathrm{op}}(t)$, $D_{\mathrm{op}}(t)$, $D_{\mathrm{cl}}(t)$, and

$$\mathbf{K} = \begin{bmatrix} -k_{\mathrm{op}} & +k_{\mathrm{cl}} & 0 & 0 \\ +k_{\mathrm{op}} & -k_{\mathrm{cl}} - k_{\mathrm{forw}} & +k_{\mathrm{back}} & 0 \\ 0 & +k_{\mathrm{forw}} & -k'_{\mathrm{cl}} - k_{\mathrm{back}} & +k'_{\mathrm{op}} \\ 0 & 0 & +k'_{\mathrm{cl}} & -k'_{\mathrm{op}} \end{bmatrix}. \tag{S10}$$

The natural state space for the vectors $\mathbf{x}(t)$ is

$$\Omega = \left\{ \mathbf{r} = \begin{bmatrix} r_1 & r_2 & \cdots & r_n \end{bmatrix}^{\mathrm{T}} \text{ s.t. } r_k \in \mathbb{R}, r_k > 0 \text{ for all } k = 1, 2, \ldots, n, \text{ and } \sum_{i=1}^{n} r_k = 1 \right\},$$

also called the probability simplex. Here, $n = 4$.

The stationary solution, i.e. satisfying $\mathbf{K}\mathbf{x}_{\mathrm{eq}} = \mathbf{0}$, is

$$\mathbf{x}_{\mathrm{eq}} = \frac{\begin{bmatrix} K_{\mathrm{back}}P & K_{\mathrm{back}} & 1 & P' \end{bmatrix}^{\mathrm{T}}}{K_{\mathrm{back}}(1+P) + (1+P')} = \frac{\begin{bmatrix} K_{\mathrm{back}}P & K_{\mathrm{back}} & 1 & P + \delta P \end{bmatrix}^{\mathrm{T}}}{(1+K_{\mathrm{back}})(1+P) + \delta P}, \tag{S11}$$

where $P = k_{\mathrm{cl}}/k_{\mathrm{op}}$ and $P' = k'_{\mathrm{cl}}/k'_{\mathrm{op}} = P(1+\delta)$ are protection factors, and $K_{\mathrm{back}} = k_{\mathrm{back}}/k_{\mathrm{forw}}$ is the equilibrium constant of the back exchange reaction. Existence of a stationary solution is always guaranteed for the model (S9) because the equations of the system (or the rows of K) are linearly dependent. This also guarantees that $\mathbf{K}$ has an eigenvalue $\lambda_0 = 0$, whose eigenvector is $\mathbf{v}_0 \propto \mathbf{x}_{\mathrm{eq}}$. From Eq. S11, it results

$$\frac{D_{\mathrm{eq}}}{H_{\mathrm{eq}}} = \frac{1}{K_{\mathrm{back}}} \left(\frac{1+P'}{1+P} \right) = \frac{1}{K_{\mathrm{back}}} \left(1 + \frac{\delta P}{1+P} \right). \tag{S12}$$

2.1 Exact solution

The solution of the GLL model (S9) can always be written in terms of the flow $\phi^t : \Omega \to \Omega$, which here is simply the matrix exponential. Its action evolves an initial condition $\mathbf{x}(0)$ as $\mathbf{x}(t) = \phi^t(\mathbf{x}(0)) = \mathrm{e}^{\mathbf{K}t}\mathbf{x}(0)$. Diagonalizing matrix $\mathbf{K}$, the solution can be written in terms of eigenvalues $\{\lambda\}$ and eigenvectors $\{\mathbf{v}_\lambda\}$ of $\mathbf{K}$ as

$$\mathbf{x}(t) = \sum_{\lambda} c_\lambda \mathrm{e}^{-\lambda t} \mathbf{v}_\lambda = \mathbf{x}_{\mathrm{eq}} + \sum_{\lambda \neq 0} c_\lambda \mathrm{e}^{-\lambda t} \mathbf{v}_\lambda,$$

where $\{c_\lambda\}$ are coefficients that depend on initial condition $\mathbf{x}(0)$ and in the last equality the equilibrium state $\mathbf{x}_{\mathrm{eq}}$ has been evidenced. $\mathbf{K}$ is a real tridiagonal matrix, i.e. it has the form

$$\begin{bmatrix} a_1 & b_1 & 0 & & & 0 \\ c_1 & a_2 & b_2 & & & \\ 0 & c_2 & \ddots & \ddots & & \\ & & \ddots & & \ddots & \\ & & & \ddots & \ddots & b_{n-1} \\ 0 & & & & c_{n-1} & a_n \end{bmatrix}. \tag{S13}$$

Because $\mathbf{K}$ (Eq. S10) is similar to a real symmetric tridiagonal matrix $\mathbf{S}$ with non-null diagonal and off-diagonal values, a theorem guarantees that all its eigenvalues are real and simple (i.e., they are all distinct and associated to a unique eigenvector).[6] The eigenvalues of $\mathbf{S}$ coincide with those of $\mathbf{K}$. If $\mathbf{S} = \mathbf{D}^{-1}\mathbf{K}\mathbf{D}$, the eigenvector $\mathbf{v}_\lambda$ of $\mathbf{K}$ is given by $\mathbf{v}_\lambda = \mathbf{D}\mathbf{u}_\lambda$, where $\mathbf{u}_\lambda$ is the eigenvector of $\mathbf{S}$ corresponding to the same eigenvalue λ. $\mathbf{S}$ can be obtained for example by a diagonal matrix $\mathbf{D} = \mathrm{diag}(d_k)$ with $d_k = \sqrt{x_{\mathrm{eq},k}}$, where the $\{x_{\mathrm{eq},k}\}$ are the components of $\mathbf{x}_{\mathrm{eq}}$ (Eq. S11). The result is

$$\mathbf{S} = \begin{bmatrix} -k_{\mathrm{op}} & +\sqrt{k_{\mathrm{op}}k_{\mathrm{cl}}} & 0 & 0 \\ +\sqrt{k_{\mathrm{op}}k_{\mathrm{cl}}} & -k_{\mathrm{cl}} - k_{\mathrm{forw}} & +\sqrt{k_{\mathrm{forw}}k_{\mathrm{back}}} & 0 \\ 0 & \sqrt{k_{\mathrm{forw}}k_{\mathrm{back}}} & -k'_{\mathrm{cl}} - k_{\mathrm{back}} & +\sqrt{k'_{\mathrm{op}}k'_{\mathrm{cl}}} \\ 0 & 0 & +\sqrt{k'_{\mathrm{op}}k'_{\mathrm{cl}}} & -k'_{\mathrm{op}} \end{bmatrix}. \tag{S14}$$

The desired eigenvalues λ_k are the roots of the characteristic polynomial $p(\lambda) = \det(\lambda\mathbf{I} - \mathbf{S})$. For a $n \times n$ real tridiagonal matrix $\mathbf{T}$ with entries named as in Eq. S13, let $\mathbf{T}_m$ the $m \times m$ ($m \leq n$) principal submatrix of $\mathbf{T}$, obtained deleting rows and columns $m+1, m+2, \ldots, n$ from $\mathbf{T}$. The polynomials $p_m(\lambda) = \det(\lambda\mathbf{I} - \mathbf{T}_m)$ satisfy the recurrence relation

$$p_m(\lambda) = (\lambda - a_m)p_{m-1}(\lambda) - b_{m-1}c_{m-1}p_{m-2}(\lambda), \tag{S15}$$

with initial conditions $p_0(\lambda) = 1$, $p_1(\lambda) = \lambda - a_1$. Because by definition $\mathbf{T} = \mathbf{T}_n$, the characteristic polynomial of $\mathbf{T}$ is $p_n(\lambda)$. Dividing Eq. S15 by $b_1 \cdots b_{n-1}$, a recursive relation for the eigenvectors is obtained:

$$u_{\lambda,j} = w_\lambda \frac{p_{j-1}(\lambda)}{b_1 \cdots b_{j-1}}, \quad j = 2, \ldots, n,$$

where $u_{\lambda,j}$ is the j-th component of the eigenvector $\mathbf{u}_\lambda$ of $\mathbf{T}$, and $u_{\lambda,1} = w_\lambda$ is determined by the normalization as

$$w_\lambda^{-1} = \sqrt{\frac{p_{n-1}(\lambda)}{(b_1 \cdots b_{n-1})^2} \prod_{\lambda' \neq \lambda} (\lambda' - \lambda)}.$$

The characteristic polynomial of $\mathbf{S}$ (Eq. S14) was computed recursively by Eq. S15:

$$p(\lambda) = \lambda\left(\lambda^3 + A\lambda^2 + B\lambda + C\right), \tag{S16}$$

where

$$\begin{aligned}
A =& k_{\text{op}} + k'_{\text{op}} + k_{\text{cl}} + k'_{\text{cl}} + k_{\text{forw}} + k_{\text{back}}, \\
B =& k_{\text{op}}k'_{\text{op}} + k_{\text{op}}k'_{\text{cl}} + k_{\text{op}}k_{\text{forw}} + k_{\text{op}}k_{\text{back}} + k'_{\text{op}}k_{\text{cl}} \\
&+ k'_{\text{op}}k_{\text{forw}} + k'_{\text{op}}k_{\text{back}} + k_{\text{cl}}k'_{\text{cl}} + k_{\text{cl}}k_{\text{back}} + k'_{\text{cl}}k_{\text{forw}}, \\
C =& k_{\text{op}}k'_{\text{op}}k_{\text{forw}} + k_{\text{op}}k'_{\text{op}}k_{\text{back}} + k_{\text{op}}k'_{\text{cl}}k_{\text{forw}} + k'_{\text{op}}k_{\text{cl}}k_{\text{back}}.
\end{aligned}$$

From Eq. S16, it is evident that one eigenvalue is $\lambda = 0$. The other three are the roots of the polynomial $\lambda^3 + A\lambda^2 + B\lambda + C$. These can be obtained transforming the equation in a depressed cubic $y^3 + py + q = 0$, upon the change of variable $\lambda = y - A/3$, which is a particular case of Cardano's method. The coefficients are

$$p = B - \frac{A^2}{3}, \quad q = \frac{2A^3}{27} - \frac{AB}{3} + C,$$

and the solutions y_k are given by $y_k = z_{+,k} + z_{-,k}$, where $z_{\pm,k}$ is the k-th root of

$$\sqrt[3]{-\frac{q}{2} \pm \sqrt{\Delta_{\text{C}}}}, \quad \text{with}\, \Delta_{\text{C}} = \left(\frac{q}{2}\right)^2 + \left(\frac{p}{3}\right)^2.$$

Because in this case eigenvalues are ensured to be real and distinct, $\Delta_{\text{C}} < 0$ and one can use the trigonometric (cosine) form of Cardano to express the solutions:

$$y_k = 2\sqrt{-\frac{p}{3}} \cos\left[\frac{1}{3} \arccos\left(\frac{3q}{2p}\sqrt{-\frac{3}{p}} - \frac{2\pi(k-1)}{3}\right)\right], \quad k = 1, 2, 3.$$

Eigenvalues can be then recovered as $\lambda_k = y_k - A/3$, and eigenvectors computed from the recursive method described above. However, the expressions obtained (in terms of the rates) result intractable unless assumptions on the rates are introduced.

2.2 Approximations

2.2.1 Steady state approximation

The steady state approximation (SSA) can be applied to the GLL model (S9) assuming that the open states H_{op} and D_{op} are short-lived intermediates that form a unique transition state.[7] This condition is satisfied if $k_{\text{cl}} \gg k_{\text{op}}$ and $k'_{\text{cl}} \gg k'_{\text{op}}$, i.e. if $P \gg 1$ and $P' \gg 1$, thus represents a generalized version of the native approximation in the Linderstrøm-Lang model. The reaction (S9) is simplified to an effective two-state reaction:[7]

$$\text{H} \underset{k_-}{\overset{k_+}{\rightleftharpoons}} \text{D}, \tag{S17}$$

where

$$k_+ = \frac{k_{\rm op} k_{\rm forw} k'_{\rm cl}}{k_{\rm cl} k_{\rm back} + k_{\rm cl} k'_{\rm cl} + k'_{\rm cl} k_{\rm forw}}, \tag{S18a}$$

$$k_- = \frac{k_{\rm cl} k_{\rm back} k'_{\rm op}}{k_{\rm cl} k_{\rm back} + k_{\rm cl} k'_{\rm cl} + k'_{\rm cl} k_{\rm forw}}. \tag{S18b}$$

The solution of the kinetics is

$$D(t) = \frac{1}{1+K} + \left(D_0 - \frac{1}{1+K}\right) \mathrm{e}^{-(k_+ + k_-)t}, \tag{S19}$$

where K is the equilibrium constant of reaction (S17), $K = k_+/k_-$, that coincides with the ratio $D_{\rm eq}/H_{\rm eq}$ of Eq. S12. One can rewrite Eqs. S18a and S18b as[7]

$$k_+ = \left[\left(\frac{H_{\rm op,eq}}{H_{\rm cl,eq}} k_{\rm forw}\right)^{-1} + (k_{\rm op})^{-1} + \left(\frac{D_{\rm eq}}{H_{\rm eq}} k'_{\rm op}\right)^{-1}\right]^{-1}, \tag{S20a}$$

$$k_- = \left[\left(\frac{D_{\rm op,eq}}{D_{\rm cl,eq}} k_{\rm back}\right)^{-1} + \left(k'_{\rm op}\right)^{-1} + \left(\frac{H_{\rm eq}}{D_{\rm eq}} k_{\rm op}\right)^{-1}\right]^{-1}. \tag{S20b}$$

Since by assumption $P \gg 1$, the equilibrium constant K can be simplified to

$$K = \frac{1+\delta}{K_{\rm back}}, \tag{S21}$$

i.e. it is independent of P and accounts for $P \neq P'$ by δ. Introducing the definitions of P, P' and K, the rates $k_\pm$ become

$$(k_+)^{-1} = \left(\frac{k_{\rm forw}}{P}\right)^{-1} + (k_{\rm op})^{-1} + \left(\frac{1+\delta}{K_{\rm back}}\right)^{-1} (k'_{\rm op})^{-1}, \tag{S22a}$$

$$(k_-)^{-1} = \left(\frac{k_{\rm back}}{P'}\right)^{-1} + \left(k'_{\rm op}\right)^{-1} + \left(\frac{1+\delta}{K_{\rm back}}\right) (k_{\rm op})^{-1}. \tag{S22b}$$

In Eqs. S22a and S22b, the first term indicates the time required to cross the transition state (which is assumed in equilibrium with the initial but not with the final state), the other two describe processes that restore equilibrium from the side of initial and final states. If crossing the barrier is the limiting factor, the first components are the longest:

$$k_+ = \frac{k_{\rm forw}}{P}, \quad k_- = \frac{k_{\rm back}}{P'},$$

which give an observed rate

$$k_{\rm obs} = \frac{k_{\rm int,mix}}{P}\left(1 - \frac{K_{\rm back}}{1+K_{\rm back}} \frac{\delta}{1+\delta}\right), \tag{S23}$$

where $k_{\text{int,mix}} = k_{\text{forw}} + k_{\text{back}}$. Conversely, in EX1, one has

$$k_{\text{obs}} = k_{\text{op}} \left(1 - \frac{1-\xi}{K+\xi} \right), \tag{S24}$$

where

$$\xi = \frac{k_{\text{op}}}{k'_{\text{op}}}, \tag{S25}$$

and K is given by Eq. S21. Eqs. S23 and S24 are formally analogous to the solutions of the Linderstrøm-Lang model, Eqs. S7a and S7b, upon substitution of k_{int} by $k_{\text{int,mix}}$ and introducing a factor that accounts for $k'_{\text{cl}} \neq k_{\text{cl}}$ and $k'_{\text{op}} \neq k_{\text{op}}$. The LL case is evidently retrieved if $K_{\text{back}} = 0$, i.e. $k_{\text{forw}} = k_{\text{int}}$ and $k_{\text{back}} = 0$.

2.2.2 Pre-equilibrium approximation

A pre-equilibrium approximation applied to the GLL model (S9) implies a separation of time scales: if motility ($k_{\text{op}} + k_{\text{cl}}$ and $k'_{\text{op}} + k'_{\text{cl}}$) is much faster than exchange ($k_{\text{int,mix}} = k_{\text{forw}} + k_{\text{back}}$), H_{op} and D_{op} can be assumed to attain instantaneous equilibrium with their closed counterparts, thus, for any t,

$$\frac{H_{\text{op}}(t)}{H_{\text{cl}}(t)} = \frac{1}{P},\ \frac{D_{\text{op}}(t)}{D_{\text{cl}}(t)} = \frac{1}{P'} \implies H_{\text{op}}(t) = \frac{H(t)}{1+P},\ D_{\text{op}}(t) = \frac{D(t)}{1+P'}.$$

Because of these relations, the study of the GLL model (S9) reduces to a two-state reaction analogous to (S17). Here, the resulting equation is

$$\begin{aligned} \dot{D}(t) =& k_{\text{forw}} H_{\text{op}}(t) - k_{\text{back}} D_{\text{op}}(t) \\ =& \frac{k_{\text{forw}}}{1+P}(1 - D(t)) - \frac{k_{\text{back}}}{1+P'} D(t) \\ =& -\frac{k_{\text{int,mix}}}{1+P} \left(1 - \frac{K_{\text{back}}}{1+K_{\text{back}}} \frac{\delta P}{1+P+\delta P} \right) D(t) + \frac{k_{\text{int,mix}}}{1+P} \frac{1}{1+K_{\text{back}}}, \end{aligned} \tag{S26}$$

hence

$$k_{\text{obs}} = \frac{k_{\text{int,mix}}}{1+P} \left(1 - \frac{K_{\text{back}}}{1+K_{\text{back}}} \frac{\delta P}{1+P+\delta P} \right). \tag{S27}$$

For $P \gg 1$, $\delta P/(1+P+\delta P) \simeq \delta/(1+\delta)$, and Eq. S27 simplifies to the EX2 case (Eq. S23).

2.2.3 No isotopic substitution effects

For the generalized Linderstrøm-Lang model that ignores the effects of isotopic substitution on stability, i.e. $k'_{\rm cl} = k_{\rm cl}$, $k'_{\rm op} = k_{\rm op}$ ($\delta = 0$),

$$\mathrm{H_{cl}} \underset{k_{\rm cl}}{\overset{k_{\rm op}}{\rightleftharpoons}} \mathrm{H_{op}} \underset{k_{\rm back}}{\overset{k_{\rm forw}}{\rightleftharpoons}} \mathrm{D_{op}} \underset{k_{\rm op}}{\overset{k_{\rm cl}}{\rightleftharpoons}} \mathrm{D_{cl}}, \tag{S28}$$

eigenvalues $\{\lambda\}$ and (non-normalized) eigenvectors $\{\mathbf{v}_\lambda\}$ satisfying $\mathbf{K}\mathbf{v}_\lambda = \lambda\mathbf{v}_\lambda$ can be explicitly written as

$$\begin{cases} \lambda_0 = 0 \\ \mathbf{v}_0 = \begin{bmatrix} PK_{\rm back} & K_{\rm back} & 1 & P \end{bmatrix}^{\rm T}, \end{cases} \tag{S29a}$$

$$\begin{cases} \lambda_1 = -(k_{\rm op} + k_{\rm cl}) \\ \mathbf{v}_1 = \begin{bmatrix} K_{\rm back} & -K_{\rm back} & -1 & 1 \end{bmatrix}^{\rm T}, \end{cases} \tag{S29b}$$

$$\begin{cases} \lambda_\pm = -\frac{1}{2}\left(\gamma \pm \sqrt{\gamma^2 - 4k_{\rm op}k_{\rm int,mix}}\right) \\ \mathbf{v}_\pm = \begin{bmatrix} -1 & -\frac{k_{\rm op}}{k_{\rm cl}} + \frac{\lambda_\pm}{k_{\rm cl}} & -\frac{k_{\rm op}}{k_{\rm cl}} - \frac{\lambda_\pm}{k_{\rm cl}} & 1 \end{bmatrix}^{\rm T}, \end{cases} \tag{S29c}$$

where $K_{\rm back} = k_{\rm back}/k_{\rm forw}$ is the equilibrium constant of the elementary back exchange reaction, $P = k_{\rm cl}/k_{\rm op}$ is the protection factor, and $\gamma = k_{\rm op} + k_{\rm cl} + k_{\rm forw} + k_{\rm back}$. The eigenvalue λ_0 is associated with the stationary solution

$$\mathbf{x}_{\rm eq} = \frac{\mathbf{v}_0}{(1 + K_{\rm back})(1 + P)}, \tag{S30}$$

which predicts an equilibrium amide deuteration $D_{\rm eq}$ that depends only on $K_{\rm back}$, that is,

$$\frac{D_{\rm eq}}{H_{\rm eq}} = \frac{1}{K_{\rm back}}. \tag{S31}$$

Eigenvalue λ_1 and eigenvector $\mathbf{v}_1$ describe no net exchange, rather encode motility, i.e. opening/closing dynamics.[1] Eigenvalues $\lambda_\pm$ and eigenvectors $\mathbf{v}_\pm$ describe exchange. It is noted that results of Eqs. S29a, S30 and S31 are formally analogous to those that hold for the GLL model (S9) for $\delta = 0$.

The SSA applied to the model (S28) gives again a two-state reaction as in (S17), with rates

$$k_+ = \frac{k_{\rm op}k_{\rm forw}}{k_{\rm cl} + k_{\rm forw} + k_{\rm back}}, \tag{S32a}$$

$$k_- = \frac{k_{\rm op}k_{\rm back}}{k_{\rm cl} + k_{\rm forw} + k_{\rm back}}. \tag{S32b}$$

In this case,

$$k_{\mathrm{obs}} = \frac{k_{\mathrm{op}} k_{\mathrm{int,mix}}}{k_{\mathrm{cl}} + k_{\mathrm{int,mix}}}, \tag{S33}$$

which is formally analogous to the native approximation of the Linderstrøm-Lang model. In the EX1 case, $k_{\mathrm{cl}} \ll k_{\mathrm{int,mix}}$, Eq. S33 reduces to

$$k_{\mathrm{obs}} = k_{\mathrm{op}}. \tag{S34}$$

For the EX2 case, $k_{\mathrm{cl}} \gg k_{\mathrm{op}}$, one finds

$$k_{\mathrm{obs}} = \frac{k_{\mathrm{int,mix}}}{P} \tag{S35}$$

Note that Eqs. S34 and S35 coincide with Eqs. S24 and S23 for $\delta = 0$ and $\xi = 1$, *cfr* Eq. S25, which are exactly the assumptions introduced the model (S28).

The pre-equilibrium approximation gives

$$k_{\mathrm{obs}} = \frac{k_{\mathrm{int,mix}}}{1 + P}, \tag{S36}$$

consistently with Eq. S27 for the case $\delta = 0$.

3 Supporting Figures

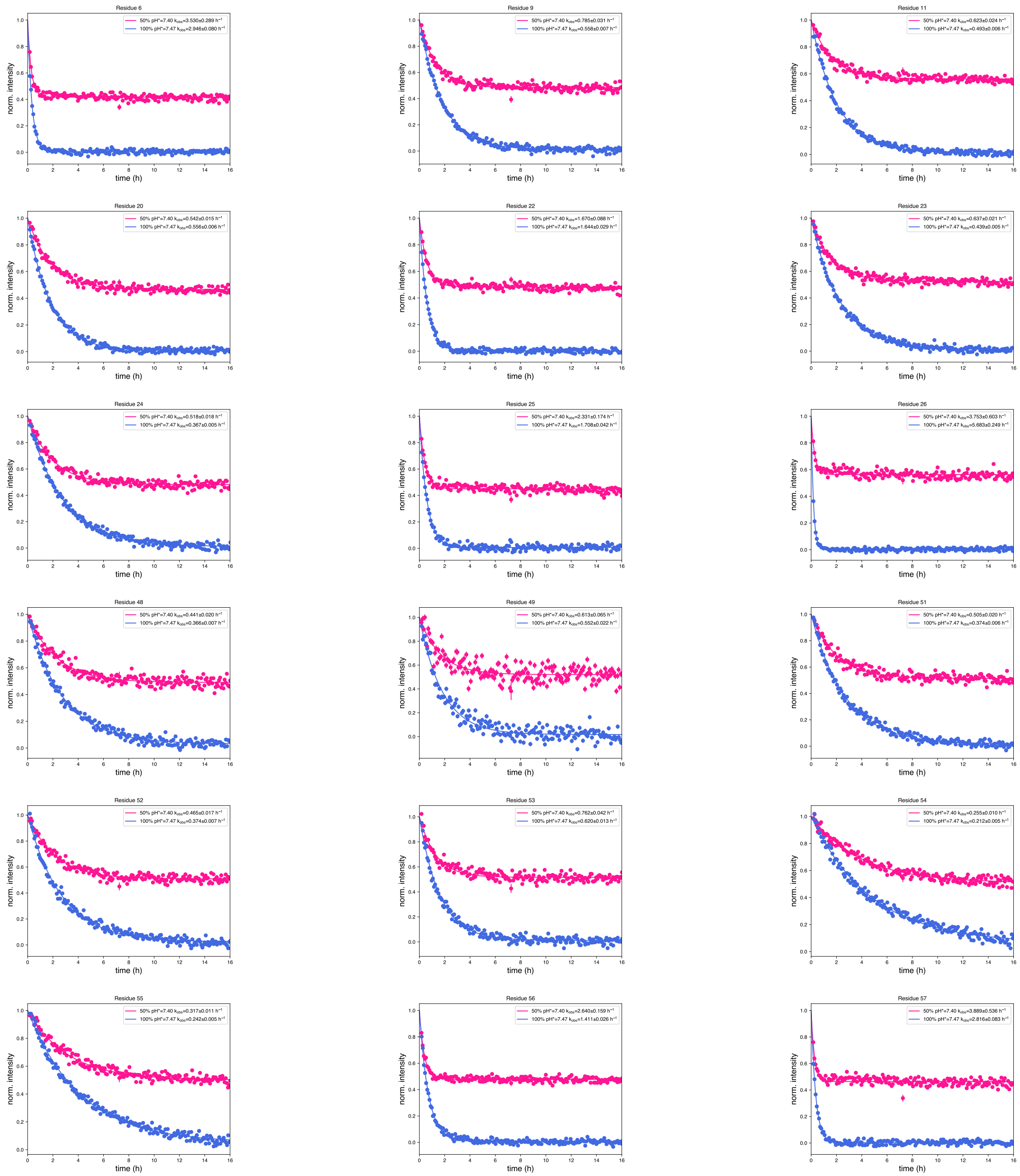

Figure S1: HDX/NMR experimental data (points) and exponential fit (line) for 18 amides of ^{15}N-DNAJB1 JD-GF-α5 F94L, in 50% D_2O at pH* = 7.40 (magenta) and 100% D_2O at pH* = 7.47 (blue).

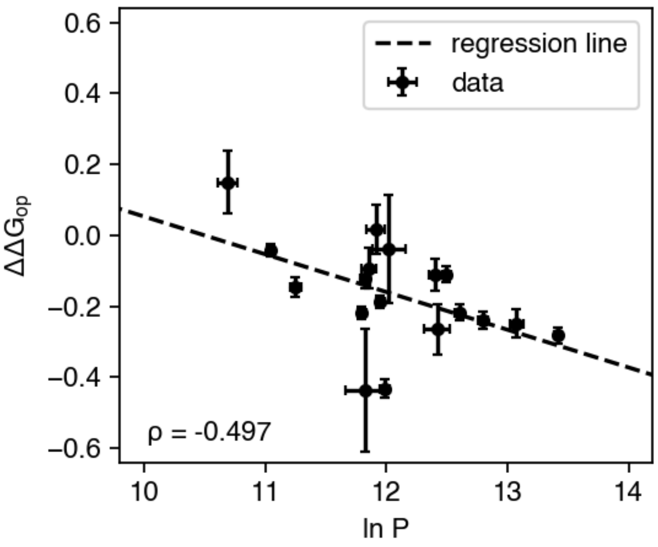

Figure S2: Moderate anti-correlation ($\rho = -0.497$) between $\Delta\Delta G_{\text{op}} = RT\ln(1+\delta)$ (Eq. 6 in main text), inferred from measurements in 50% D_2O, and $\ln P$, measured in 100% D_2O.

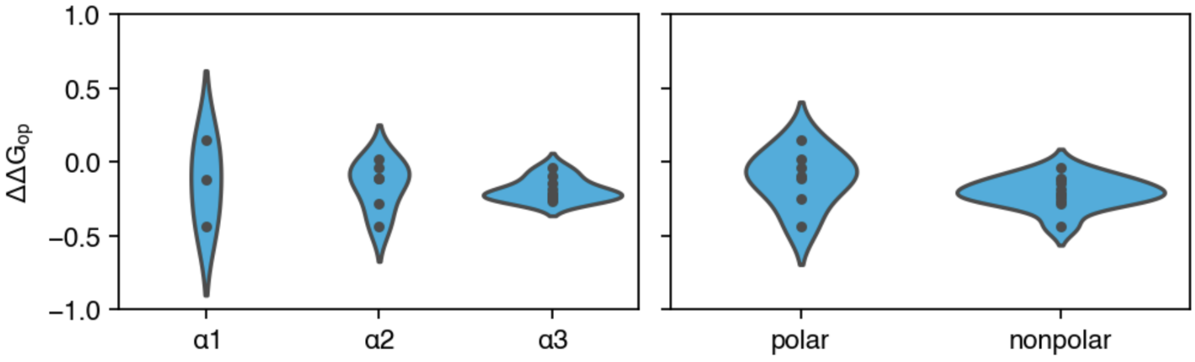

Figure S3: Violin plot of the kernel density estimated distributions of $\Delta\Delta G_{\mathrm{op}}$, with residues represented as points and grouped by: (left) helix, (right) residue type.

References

(1) Hvidt, A.; Nielsen, S. O. Hydrogen exchange in proteins. *Advances in protein chemistry* **1966**, *21*, 287–386.

(2) Hilser, V. J.; Freire, E. Structure-based calculation of the equilibrium folding pathway of proteins. Correlation with hydrogen exchange protection factors. *Journal of molecular biology* **1996**, *262*, 756–772.

(3) Bai, Y.; Milne, J. S.; Mayne, L.; Englander, S. W. Primary structure effects on peptide group hydrogen exchange. *Proteins: Structure, Function, and Bioinformatics* **1993**, *17*, 75–86.

(4) Connelly, G. P.; Bai, Y.; Jeng, M.-F.; Englander, S. W. Isotope effects in peptide group hydrogen exchange. *Proteins: Structure, Function, and Bioinformatics* **1993**, *17*, 87–92.

(5) Nguyen, D.; Mayne, L.; Phillips, M. C.; Walter Englander, S. Reference parameters for protein hydrogen exchange rates. *Journal of the American Society for Mass Spectrometry* **2018**, *29*, 1936–1939.

(6) Parlett, B. N. *The symmetric eigenvalue problem*; SIAM, 1998.

(7) Kurzynski, M. *The Thermodynamic Machinery of Life*; Springer Berlin Heidelberg, 2006.
